# Plausible and Proper Multiple-Choice Items for Diagnostic Classification

**DOI:** 10.1017/psy.2025.10074

**Published:** 2025-12-19

**Authors:** Chia-Yi Chiu, Hans Friedrich Köhn, Yu Wang

**Affiliations:** 1 Human Development, Teachers College at Columbia University, USA; 2 Psychology, University of Illinois at Urbana–Champaign, USA; 3 Educational Testing Service, Princeton, USA

**Keywords:** cognitive diagnosis, MC-DINA model, MC-NPC method, multiple-choice items, proper and plausible MC items

## Abstract

The multiple-choice (MC) item format has been adapted to the cognitive diagnosis (CD) framework. Early approaches simply dichotomized the responses and analyzed them with a CD model for binary responses. Obviously, this strategy cannot exploit the additional diagnostic information provided by MC items. De la Torre’s (2009, *Applied Psychological Measurement*, 33, 163–183) MC-DINA model was the first for the explicit analysis of MC items. However, the q-vectors of the distractors were constrained to be nested within the key and each other, which imposes serious restrictions on item development. Relaxing the nestedness-constraint, comes at a price. First, distractors may become redundant: they do not improve the classification of examinees beyond the response options already available for an item. Second, undesirable diagnostic ambiguity can arise from distractors that are equally likely to be chosen by an examinee, but have distinct attribute profiles pointing at different diagnostic classifications. In this article, two criteria, *plausible* and *proper*, are developed for detecting these problematic cases. Two theorems that permit for the detection and amendment of improper and implausible items are presented. An R function serving this purpose is used in several practical applications. Results of simulation studies and real data analysis are also reported.

## 1 Introduction

The multiple-choice (MC) item format has been implemented in educational assessments that are used across diverse content domains. MC items comprise two components: the stem prepares examinees for the test questions in providing the context and a motivating narrative; and the collection of response options contains the correct answer, called the “key,” and several incorrect alternatives, the “distractors.” Different from the dichotomous response format, MC items using the polytomous response format allegedly permit for collecting richer diagnostic information, while examinees need to spend less time on recording their answers. MC items are also less vulnerable to subjective scoring (cf. Burton et al., 1991). In summary, the economy of MC items is likely one of the reasons for their persistent popularity not just in educational testing.

The MC item format has been adapted to accommodate also the cognitive diagnosis (CD) framework in educational measurement. Within CD, ability—or competence—in a curricular knowledge domain is perceived as a composite of cognitive skills called “attributes.” CD-based tests consist of items that require for a correct response mastery of different attributes. From the item responses, examinees’ ability can be inferred and evaluated in terms of attributes mastered and those needing study.

The polytomous items response format has also received considerable attention in the field of knowledge space theory (KST; for general presentations of KST, cf. Doignon and Falmagne (2017) and Falmagne and Doignon (2011); for the specific connection between KST and CD, cf., e.g., Heller et al., 2015, 2016). Lucid discussions elaborating the sophisticated theoretical foundations of polytomous KST models and their connection with items using the polytomous response format in CD can be found, for example, in Stefanutti et al. (2020) and Stefanutti et al. (2023).

Early approaches to analyzing MC items within the CD framework lacked sophistication such that the MC responses were simply dichotomized in scoring the key as 1 and the distractors as 0 (e.g., Lee et al., 2011; Templin & Henson, 2006). The dichotomized responses were then analyzed using one of the CD models—diagnostic classification model (DCM) hereafter—for binary responses. De la Torre’s (2009) Multiple-Choice Deterministic Inputs, Noisy “And” Gate (MC-DINA) model was the first DCM for analyzing MC items in considering the distractors explicitly as potential sources of diagnostic information. Recall that in case of the DINA model, a binary item can only discriminate between examinees, who have mastered all required attributes and those who fail one or more of these attributes. Extending the model to accommodate the MC item format is expected to increase the classification accuracy, as examinees can then be assigned to more than two groups. The implementation of the MC item format in de la Torre’s (2009) MC-DINA model, however, requires imposing the hierarchical structural constraint such that the attribute profiles of the distractors are contained within that of the key and within one another. This property is typically referred to as “nestedness.” Obviously, this nestedness requirement puts an extra burden on the test developer, as the options for item building are more constrained. In fact, later adaptations of the MC item format to CD—including the current implementation of de la Torre’s (2009) MC-DINA in the R package GDINA (Ma & de la Torre, 2020)—have abandoned the nestedness requirement (e.g., DiBello et al., 2015; Ozaki, 2015; Wang et al., 2023).

The relaxation of the nestedness-condition, however, comes at a price. First, distractors may become redundant; that is, they do not contribute to any further diagnostic differentiation between examinees. The inclusion of redundant distractors in a test wastes valuable item space and may increase unsystematic error variance. Redundancy among distractors, however, may be difficult to detect. Second, undesirable diagnostic ambiguity can arise from distractors that are equally likely to be chosen by an examinee, but have distinct attribute profiles pointing at different diagnostic classifications. Recall that in the case of the “original” MC-DINA model (de la Torre, 2009) the restriction that all distractors must be nested within each other avoids these issues. Comprehensive explanations and illustrations of these complex concepts are provided in Section 3.

Ozaki (2015) appears to be the first who observed this specific form of diagnostic ambiguity arising from relaxing the nestedness condition. As a remedy, he proposed “structured MC-DINA models” (MC-S-DINA) for pinpointing (and resolving) diagnostic ambiguity through augmenting the model with parameters that explicitly target potentially ill-separated effects of differential attribute mastery. The parameters of the MC-S-DINA models were estimated with MCMC.

In this article, a very different approach is presented to deal with diagnostic ambiguity and diagnostic redundancy as they might arise from removing the nestedness condition. Two criteria, *plausible* and *proper*, are developed that conceptualize these two phenomena as resulting from the inadequate specification of the distractors of an MC item. An item is said to be *plausible* if its distractors are not redundant. A redundant distractor is one that does not improve the classification of examinees beyond the response options already available for a given item. An item is called *proper* if its distractors allow for the unambiguous identification of an examinee’s ideal response. Two theorems are presented that serve as the theoretical foundation of the concepts of “plausible” and “proper” items.

The next section briefly reviews essential CD concepts and their adaptation to accommodate MC items like the MC-DINA model and the nonparametric classification method for MC items (MC-NPC). Section 3 presents the theory of “plausible” and “proper” items. Results of simulation studies conducted for the empirical evaluation of these two concepts are reported in Section 4, followed by two practical applications described in Section 5. The discussion section concludes with a summary of the main findings and some thoughts about potential limitations and future research avenues.

## 2 Review of key technical concepts

### 2.1 Cognitive diagnosis

DCMs for CD, a formative assessment framework in educational measurement, describe ability in a given knowledge domain as a composite of *K* cognitive skills—henceforth: “attributes”—that a student has mastered or not (DiBello et al., 2007; Haberman & von Davier, 2007; Leighton & Gierl, 2007; Nichols et al., 1995; Rupp et al., 2010; Sessoms & Henson, 2018; Tatsuoka, 2009; von Daver & Lee, 2019). Attribute mastery is recorded as a *K*-dimensional binary vector 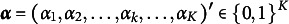 . Distinct attribute profiles 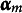 identify different classes of proficiency 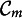 , 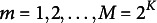 (provided the attributes are not hierarchically organized). (The terms profile and vector are used interchangeably here.) The primary task of CD is to assign students to one of these *M* classes based on their performance in a test that targets proficiency in the knowledge domain in question. Said differently, examinees’ individual attribute vectors 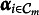 must be estimated ( 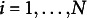 is the examinee index; for brevity, 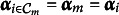 is often used, depending on the context).

CD items require mastery of domain-specific attributes for a correct response. Similar to examinees, CD items are characterized by *K*-dimensional attribute profiles 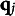 , with entries 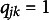 if a correct answer requires mastery of the 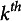 attribute 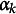 , and 0 otherwise ( 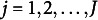 is the item index). (Notice that the zero vector is not admissible; thus, there are at most 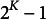 legitimate item–attribute profiles.)

The collection of the item–attribute profiles of a CD assessment forms its Q-matrix 
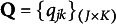
 (Tatsuoka, 1985) that establishes the associations between items and attributes. The Q-matrix of a test must be known and it must be complete. A Q-matrix is said to be complete if its specific composition can guarantee the identifiability of all realizable proficiency classes among examinees (Chiu et al., 2009; Köhn & Chiu, 2016, 2017, 2019, 2021). Q-completeness is the key requirement for the identifiability of DCMs (cf. Gu & Xu, 2021).

### 2.2 The MC-DINA model

The intuitive appeal of the simple conceptual elegance of the Deterministic Inputs, Noisy “AND” Gate (DINA) model (Haertel, 1989; Junker & Sijtsma, 2001; Macready & Dayton, 1977) is presumably the reason why the DINA model is arguably one of the most popular DCMs. The DINA is a conjunctive model, as the probability of a correct response is maximal only if an examinee has mastered all attributes required for a given item. Thus, each DINA item generates a bi-partition of the 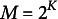 proficiency classes of the latent attribute space into groups of examinees who have mastered the attributes required for said item as opposed to those who have not. The item response function (IRF) of the DINA model is 

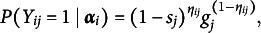

where 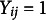 denotes the correct response to item *j* (otherwise, 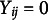 ); 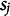 and 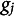 are slipping and guessing parameters, respectively, subject to 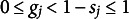 ; the ideal response (or conjunction parameter) 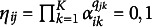 indicates whether examinee *i* has mastered all attributes required by item *j*.

The MC-DINA model was proposed by de la Torre (2009) as an extension of the DINA model to accommodate MC items. Recall that each DINA item results in a bi-partition of the latent attribute space. In contrast, an MC item partitions the latent attribute space into a number of proficiency classes that is proportional to those of coded response options, thereby purportedly increasing the accuracy of examinee classification. (A response option is said to be “coded” or “cognitively based” if it is linked to an item attribute vector 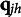 specifying the attribute requirements for an examinee who endorses this option; terminology and notation follow de la Torre (2009)). In case of the MC-DINA model, the polytomous response to item *j* is denoted as the random variable 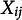 , with the response options indexed by 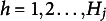 . Let 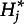 denote the number of coded options. Since not all options are coded, 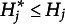 . “Non-coded” response options like “none of these” or “all of the above” are not associated with a specific attribute vector. Hence, as a convention, their item attribute vectors are written as a *K*-dimensional null vector, 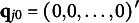 . The key always has the largest number of attributes; the attribute vectors of the coded response options must be nested within the q-vector of the key. Their attribute vectors must also be hierarchically nested within each other such that they form an ordinal scale with the key at the top. Notice that the IRF of the MC-DINA model is not provided in de la Torre’s (2009) original publication.

### 2.3 Removing the nestedness condition

As mentioned earlier, the nestedness requirement places an extra burden on test developers, as it constrains the options for item construction. To overcome this limitation, later adaptations of the MC item format for CD have abandoned the nestedness requirement—including the current implementation of de la Torre’s (2009) MC-DINA in the R package GDINA (Ma & de la Torre, 2020). Other examples include Ozaki (2015), DiBello et al. (2015), and Wang et al. (2023).

The latter, in introducing their nonparametric classification method for MC items (MC-NPC), were likely the first to provide a full formal treatment of MC items in cognitive diagnostic assessments without nestedness constraints on the attribute vectors of stems and distractors. MC-NPC is an adaptation of the nonparametric classification (NPC) method (Chiu & Douglas, 2013) for the MC item format under the DINA model. The term “nonparametric” here means that NPC methods do not rely on parametric estimation of examinees’ proficiency class membership. Instead, they use a distance-based algorithm on the observed item responses: an examinee’s response vector is compared with each of the ideal response vectors of the *M* realizable proficiency classes, and classification is based on the closest match.

When the q-vectors of the coded response options are no longer required to be nested within each other and the q-vector of the key, the linear ordering of the options is lost. This changes the scale of the item response options from ordinal to nominal—a significant conceptual departure from de la Torre’s (2009) original MC-DINA and one that necessitates several adjustments in notation:

1. The original MC-DINA response option index, 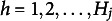 , is replaced by the index 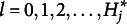 .
2. The notation for the q-vector 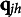 is changed to one involving the index *l*: 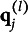 .
3. All non-coded response options are indexed as 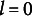 , having q-vectors 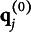 .
4. The key is now indexed as 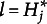 ; thus, 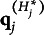 .
5. The indices 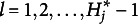 are assigned to the remaining coded response options.
6. Let 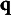 and 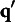 denote the q-vectors of distinct coded response options. If 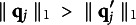 , then 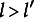 so that the notation becomes 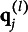 and 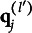 (and vice versa; 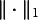 denotes the 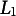 norm). If 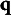 and 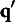 are of the same length, then the response options are indexed based on their evaluation in lexicographic order—that is, 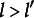 if the position of the first non-zero entry in 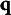 precedes that in 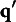 , and vice versa. Ties—both q-vectors share the position of the first non-zero entry—are ignored and the evaluation is based on the first position with distinct entries; such a position can always be identified because all coded response option q-vectors must be distinct. Formally, define the set 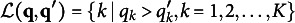 . Notice that 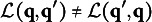 due to the evaluation of 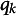 and 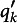 in lexicographic order. If 
(1)

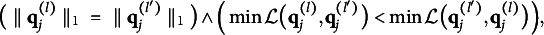

  then 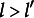 .

After the indices *l* of the item response options have been determined, the ideal response 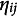 of examinee *i* to item *j* can be computed 
(2)

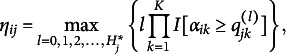

where 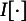 denotes the indicator function. Then, in using Equation (2), the IRF of the MC-DINA model, with the nestedness condition removed, can be expressed as 
(3)

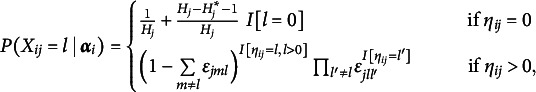

where 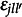 is the probability that the observed response level *l* disagrees with the ideal response level 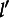 . (Because the manifest and ideal item responses, *X* and 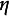 , now have more than two levels, addressing potential discrepancies between *X* and 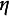 as “slips” and “guesses” appears inadequate, given the complexity of the MC setting involving multiple item parameters. The more general term “perturbation” should be preferred whenever observed and ideal responses disagree.) Typically, slipping and guessing are constrained to be less than 0.5 (otherwise, an individual mastering none of the attributes would have a probability greater than 0.5 to provide the correct answer). Of course, if there are more than two perturbation terms, then the desirable property is that 
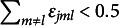
 . An examinee with 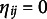 is not “attracted” (de la Torre, 2009) to any of the coded response options. Instead, said examinee is assumed to pick one of the response options at random. If 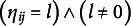 , then examinee *i* is supposed to choose the coded response option 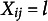 with high probability; still, alternative response options may be chosen with non-zero probability. A comprehensive example illustrating the rationale underlying Equations (2) and (3) can be found on pp. 196–198 of Wang et al. (2023).

Please notice that, henceforth, the term “MC-DINA model” refers to the CDM defined in Equations (2) and (3) presented earlier. If de la Torre’s (2009) MC-DINA model is meant, then explicit reference is made to “de la Torre’s MC-DINA model.”

## 3 The theory of plausible and proper multiple-choice items

Recall that the nestedness condition imposed on the key and coded response options can create significant difficulties in test construction, simply because the number of such nested coded response options is limited. As a less restrictive alternative, two concepts—plausible and proper—are proposed.

### 3.1 Plausible multiple-choice items

An item is said to be *plausible* if its distractors are not redundant. A redundant distractor is one that does not improve the classification of examinees beyond the information provided by the existing response options for a given item.

Here is an illustration of this concept, using an item from a test on introductory statistics:

- “Consider the dataset: 4, 6, 8, 10, 12. What is the sample variance of these data?”

Answering this item correctly requires mastery of two skills:

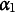
 : “Calculate squared deviations from the mean.”

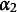
 : “Calculate sample variance by dividing sum of squared deviations by 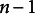 .”

Notice that the entire test is designed to measure three attributes; the third one is 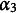 : “Understand the relationship between variance and standard deviation (variance = SD 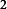 ).” The response options for this MC item consisting of the key and three distractors are reported in Table 1. Recall that for MC items, once the nestedness condition is removed, the response categories are assigned levels based on the rationale described in the previous section and summarized in Equation (1). The assigned levels of the four response options are shown in the column “Level.”

**Table 1:** Example: An MC item from an introductory statistics test

						q-vector		
Label		Answer		Level		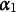	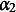	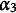
Key		10		4		0	1	1
Distractor 1		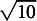		3		1	1	1
Distractor 2		12.5		2		0	0	1
Distractor 3		8		1		0	1	0

There are 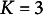 attributes; hence, there are 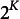 possible attribute profiles (i.e., proficiency classes). The associated ideal responses are computed using Equation (2). The complete set of 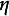 -scores for all proficiency classes is reported in Table 2 below. Notice that among the ideal responses there is a gap: 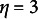 is not identified based on the information provided by the key and the coded distractors. More to the point, the distractor with level 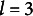 is ineffective, or redundant, because it does not contribute any information to identify its corresponding ideal response. This feature makes the item implausible. The formal definition of a “plausible” distractor is as follows.

**Table 2:** Ideal responses to the introductory statistics MC item

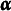	(000)	(100)	(010)	(001)	(110)	(101)	(011)	(111)
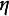	0	0	1	2	1	2	4	4

Definition 1. Suppose item *j* conforms to the MC-DINA model. Further, suppose that it has 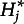 coded options. Let 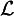 be the latent space of all realizable attribute profiles. Item *j* is said to be plausible if 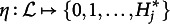 is surjective.

According to the definition, an item is plausible if its distractors allow examinees to be classified into 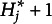 groups. Recall that the example MC item from the statistics test was implausible because the response options allowed classification into only 
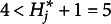
 proficiency classes. However, this item can be “repaired” to become plausible by modifying the distractors such that Distractor 1 is deleted, which results in ideal responses identical to those in Table 2 except that 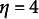 is replaced by 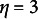 because now 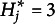 . Of course, other modifications are possible that do not involve deleting distractors, but would instead require modifying the q-vectors of the distractos, which would essentially change the content of the item.

For succinctness and technical precision, some additional notation is introduced to formulate Theorem 1. The relation of “nestedness” is denoted by “ 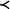 .” Specifically, for vectors 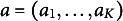 and 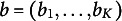 , write 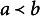 if and only if 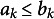 for all *k*, with 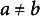 . Similarly, 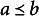 if and only if 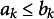 for all *k*. Without loss of generality, suppose all the coded options have been rearranged according to the rule described in Section 2.3. For item *j*, let 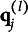 denote the q-vector of the *l*th coded option. Using this notation, Theorem 1 establishes a criterion for identifying plausible MC items.

Theorem 1. Suppose item *j* conforms to the MC-DINA model. Then, item *j* is plausible if and only if 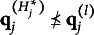 for all 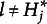 .

Verbally stated, Item *j* is plausible if and only if the q-vector of its key is not nested within any of the q-vectors of its distractors. The proof of Theorem 1 is presented in Appendix A.

Corollary 1. Suppose item *j* conforms to the MC-DINA model. If item *j* is plausible, then no coded distractor has q-vector 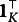 .

The corollary is an immediate consequence of the theorem and has significant practical value. If a coded distractor were to have q-vector 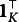 , then 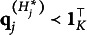 must be true, implying that item *j* is not plausible. Thus, no such coded distractor can exist.

Theorem 1 and Corollary 1 allow for a more detailed analysis of the example MC item from the introductory statistics test. In particular, since the q-vector of Distractor 1 is 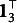 , the item is immediately identified as implausible by Corollary 1.

### 3.2 Proper multiple-choice items

An item is called *proper* if it allows for the unambiguous identification of an examinee’s ideal response. Notice that in case of de la Torre’s MC-DINA model, the restriction that all distractors must be nested within each other prevents such ambiguity.

Consider an example adapted from a binary item used by Tatsuoka (1984) as part of the test with which she collected her famous fraction-subtraction data: 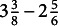 . Köhn, Chiu, Oluwalana, Kim, and Wang (2024) showed that Tatsuoka’s (1984) test involves four skills:

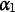
 : “Find a common denominator.”

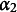
 : “Borrow from the integer.”

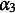
 : “Apply subtraction to integer and fraction parts separately.”

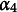
 : “Convert a fraction to a whole number.”

For the purpose of demonstrating the concept of a proper item, this binary item has been reformulated as an MC item, where the key requires mastery of the first three attributes, 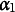 , 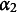 , and 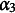 . Here are the q-vectors of the MC version of 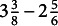 : 

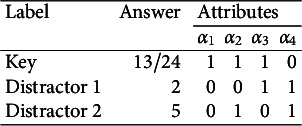

From the four attributes, 16 examinee proficiency classes can be identified ranging in attribute profiles from (0000), (1000), (0100), 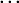 to (1111). The item key separates proficiency classes (1110) and (1111) from all other classes. Recall that, according to de la Torre’s (2009) theory, individuals in proficiency class 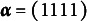 should choose only the key and none of the two distractors although both require 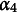 , which individuals in (1111) have mastered. Examinees who master 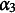 and 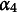 but not all four attributes—that is, proficiency classes 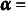 (0011), (1011), and (0111)—should choose Distractor 1 due to the following reasoning: 
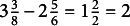
 . Concretely, Distractor 1 distinguishes 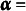 (0011), (1011), and (0111) from all remaining proficiency classes. By contrast, examinees who master 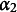 and 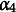 but not all four attributes are likely to select Distractor 2, reasoning that 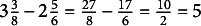 . More specifically, Distractor 2 distinguishes 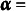 (0101), (1101), and (0111) from all remaining proficiency classes.

Diagnostic ambiguity arises because individuals in proficiency class 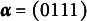 may choose either Distractor 1 or Distractor 2. In other words, the current set of response options does not allow members of proficiency class (0111) to be identified unambiguously.

The concept of proper items serves to regulate and prevent such ambiguity. It is defined as follows.

Definition 2. Suppose item *j* conforms to the MC-DINA model. Item *j* is said to be proper if for every 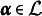 and 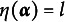 , 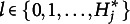 , it is true that either

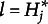

  or

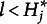
 , subject to the constraint that if there exists an 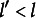 such that 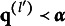 , then 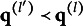 for all 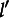 must hold.

Now, let 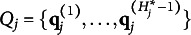 be a set that includes the q-vectors of all the coded distractors. The next theorem presents a rule for the immediate detection of improper items without having to evaluate 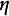 for all 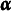 following the steps as implied by Definition 2.

Theorem 2. Suppose item *j* conforms to the MC-DINA model. Item *j* is proper if and only if for each pair of its coded distractors with q-vectors 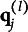 and 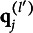 in 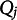 , it is true that 
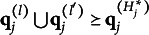
 or 
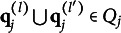
 .

Verbally stated, Item *j* is proper if and only if the q-vector of the key is equal to or nested within the union of the q-vectors of two distractors, or the union of the q-vectors of two distractors is itself a distractor. The proof of Theorem 2 is presented in Appendix A.

Theorem 2 also points at a possibility how to repair the improper MC item from Tatsuoka’s (1984) fraction-subtraction test. Concretely, the item becomes proper if a third distractor is added, whose q-vector is the union of Distractors 1 and 2, 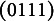 , thereby resolving the diagnostic ambiguity: 

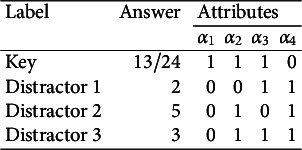



Indeed, Distractor 3 “does the trick,” as it isolates proficiency class 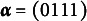 . Members of this class may reason that 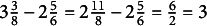 .

As a concluding remark, notice that a proper item may or may not be plausible. Said differently, plausible and proper do not condition each other. Consider these two simple examples:

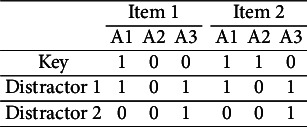



Both of the items are proper; however, Item 1 is not plausible, but Item 2 is.

## 4 Simulation studies

Two simulation studies were conducted to investigate the impact of implausible and improper MC items on the classification of examinees. Synthetic responses were generated for MC assessments that were “contaminated” with an increasing number of implausible or improper items. As noted earlier, examinee classification is only affected by these two item types when the nestedness constraint on the key and the distractors is removed. This condition applies to the MC-DINA model and the MC-NPC method, which were therefore used side-by-side for classifying examinees. Because MC-DINA and MC-NPC are known to differ in performance depending on sample size—with MC-NPC typically outperforming the parametric estimation of MC-DINA when samples are small—both methods were evaluated across a broad range of sample sizes: 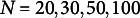 , and 300. This design allowed for a fair assessment of the relative performance of the two methods under varying conditions of data availability.

### 4.1 Study I: The effect of limitedly plausible MC items on the classification of examinees

#### 4.1.1 Design

The two major experimental factors to be manipulated were sample size and “contamination” level—that is, the number of items in the test that were of limited plausibility (NU). The five levels of sample size, 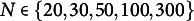 , were completely crossed with the “contamination” levels of 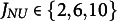 resulting in 15 experimental cells. Across all cells, the number of items was fixed at 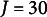 , that of attributes at 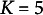 , and the number of response options for all items at 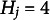 or 5.

Random Q-matrices containing only plausible items—denoted as 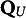 —were generated using the random multi-step procedure developed by Wang et al. (2023):

1. The q-vectors of the keys were randomly generated such that they formed a Q-matrix which conformed to the completeness condition for the DINA model (cf. Chiu et al., 2009).
2. For each key, the number of coded distractors was determined randomly in choosing from the set 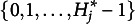 .
3. For each coded distractor, the q-vector was selected from among the 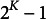 admissible item attribute patterns subject to the constraint that they were proper and plausible (i.e., allowed for the non-ambiguous identification of an examinee’s ideal response, and were non-redundant regarding examinee classification, respectively).
4. For each item, the key and the coded distractors were randomly assigned to 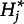 positions among the total of 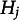 positions (recall that non-coded distractors do not have q-vector entries in the Q-matrix).

For each experimental cell, 10 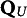 were generated and duplicated. Each of the 10 Q-matrices in the duplicated set was altered such that 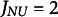 , 6, or 10 of the plausible items were turned into NU ones resulting in 10 Q-matrices 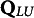 . As an illustration, a pair of contaminated and uncontaminated Q-matrices containing only 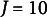 MC items (due to space restrictions) is shown in Table 3. Assume contamination level 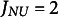 . The q-vector of the distractor in Item 3 was changed from (01001) to (11100), and that of the first distractor in Item 10 was changed from (00011) to (10111). These changes turned 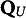 in the left panel to 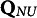 in the right panel, with the number of coded options remaining unchanged. (Notice that, in following the convention, among the q-vectors with the same item identification number, the first one is the key.)

**Table 3:** Example: A Q-matrix containing plausible items (left panel) and its altered correspondent containing two implausible items (underlain in yellow)

		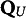						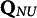				
Item	Option	A1	A2	A3	A4	A5		A1	A2	A3	A4	A5
1	3	1	0	0	0	0		1	0	0	0	0
2	1	0	1	0	0	0		0	1	0	0	0
3	3	0	0	1	0	0		0	0	1	0	0
3	1	0	1	0	0	1		1	1	1	0	0
4	4	0	0	0	1	0		0	0	0	1	0
5	3	0	0	0	0	1		0	0	0	0	1
6	2	0	1	1	1	0		0	1	1	1	0
6	1	0	0	0	0	1		0	0	0	0	1
7	4	1	1	0	1	0		1	1	0	1	0
7	3	0	1	0	1	0		0	1	0	1	0
7	1	0	0	0	1	0		0	0	0	1	0
8	4	1	0	1	1	1		1	0	1	1	1
8	2	0	1	1	1	0		0	1	1	1	0
9	3	0	0	0	1	1		0	0	0	1	1
9	4	1	0	0	0	0		1	0	0	0	0
10	3	1	0	1	0	0		1	0	1	0	0
10	1	0	0	0	1	1		1	0	1	1	1
10	2	0	0	0	1	0		0	0	0	1	0

At each of the three contamination levels, the 10 uncontaminated Q-matrices 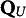 were contrasted with 10 contaminated Q-matrices 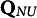 . The motivation for this elaborate procedure for devising random Q-matrices was to control for potential bias in the composition of the Q-matrix that can mask the effect of the experimental factors.

Recall the three contamination levels, 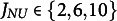 , were completely crossed with the five levels of sample size, 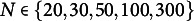 , resulting in 15 experimental cells. For each cell, 20 random Q-matrices (10 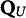 and 10 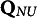 ) were generated; each random Q-matrix was used to generate 20 replicated datasets, resulting in a total of 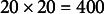 datasets per experimental cell.

A few further technical explanations might be helpful. First, to control for confounding of the impact of NU-items on classification rates with that of improper items, all items were proper. Second, to rule out the possibility that differences in classification rates might be caused by different numbers of coded options, the latter was fixed for the two types of Q-matrices. Third, examinee attribute profiles were generated based on the multivariate normal threshold model (Chiu, et al., 2009), with variances equal to 1 and covariances sampled from Unif(0.3, 0.5). Fourth, following the design in Wang et al. (2023), the probability that an examinee in group 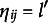 was attracted by response level 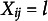 of item *j*, 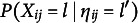 , was controlled by first generating a parameter 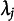 from 
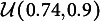
 for each item *j*. The probability 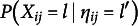 was then determined as 

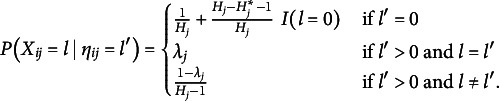



#### 4.1.2 Criteria of performance assessment

The accuracy of MC-NPC and MC-DINA in classifying examinees was quantified by the mean pattern-wise agreement rate (PAR) defined as 

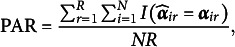

where 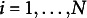 and 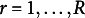 denote the examinee and replication index, respectively. For each condition, PAR quantifies the proportion of agreement between examinees’ true attribute profiles, 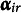 , and their estimates, 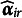 .

#### 4.1.3 Results

Tables 4 and 5 report the PAR values for MC-NPC and MC-DINA, when 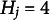 and 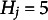 , respectively. The findings for both tables are very similar; hence, they are summarized together. First, notice that for the MC-NPC method, the PAR values in the first column 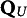 (i.e., Q-matrices containing plausible items only) are almost identical across the three cells, given a particular sample-size layer—a nice empirical demonstration of the high reliability of the MC-NPC classification. Second, notice that this stability, not too surprising, cannot be observed for the PAR values in the second column 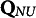 (i.e., Q-matrices contaminated with different numbers of limitedly plausible items): the more limitedly plausible items a Q-matrix contains, the more the PAR values deteriorate. Third, the columns under the header “ 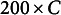 Replications” require an explanation. In a substantial number of the 200 replicated datasets, the MC-DINA algorithm encountered convergence issues such that the estimation process was disrupted and no classification result could be obtained; the proportions of these datasets are reported in parentheses (cf. columns “(C)”). In an attempt to accommodate these difficulties of MC-DINA, which are likely owed to the small sample size, the PAR values for MC-NPC were re-calculated only for these replications where MC-DINA converged. As was observed earlier, the PAR values in the 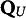 columns are stable within a sample size bracket for MC-NPC and MC-DINA. Both methods are sensitive to an increase in the number of limitedly plausible items, as it is accompanied by a decline in the PAR values. In general, the MC-NPC method clearly outperforms the MC-DINA algorithm.

**Table 4:** The effect of implausible items on the average PAR values obtained from MC-NPC and MC-DINA when 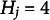

		200 replications			200 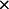 C replications				
		MC-NPC			MC-NPC			MC-DINA	
*N*	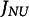	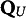	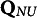		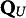	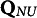		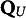 (C)	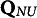 (C)
20	2	0.80	0.77		0.82	0.80		0.76 (0.14)	0.77 (0.13)
	6	0.78	0.72		0.80	0.72		0.72 (0.15)	0.69 (0.12)
	10	0.79	0.65		0.81	0.66		0.75 (0.19)	0.58 (0.08)
30	2	0.79	0.77		0.78	0.78		0.73 (0.55)	0.74 (0.41)
	6	0.79	0.74		0.79	0.74		0.77 (0.50)	0.69 (0.39)
	10	0.78	0.66		0.77	0.67		0.74 (0.59)	0.60 (0.30)
50	2	0.79	0.77		0.79	0.78		0.75 (0.89)	0.73 (0.77)
	6	0.79	0.73		0.79	0.73		0.75 (0.91)	0.69 (0.75)
	10	0.78	0.67		0.79	0.67		0.74 (0.92)	0.61 (0.67)
100	2	0.78	0.76		0.78	0.76		0.77 (0.99)	0.75 (0.98)
	6	0.78	0.73		0.78	0.73		0.76 (0.99)	0.72 (0.99)
	10	0.76	0.66		0.76	0.65		0.73 (0.97)	0.63 (0.87)
300	2	0.79	0.78		0.79	0.78		0.80 (1.00)	0.79 (1.00)
	6	0.79	0.72		0.79	0.73		0.80 (1.00)	0.76 (0.97)
	10	0.79	0.67		0.79	0.67		0.80 (1.00)	0.74 (1.00)

*Note*: “U” indicates the results for Q-matrices containing only plausible distractors. “NU” indicates the results for Q-matrices containing 2, 6, or 10 implausible distractors. “C” refers to the proportion of replications where the MC-DINA algorithm converged.

**Table 5:** The effect of implausible items on the average PAR values obtained from MC-NPC and MC-DINA when 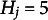

		200 Replications			200 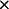 C Replications				
		MC-NPC			MC-NPC			MC-DINA	
*N*	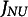	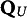	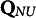		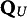	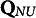		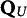 (C)	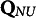 (C)
20	2	0.84	0.83		0.78	0.83		0.75 (0.01)	0.75 (0.01)
	6	0.85	0.77		0.80	0.93		0.71 (0.02)	0.82 (0.01)
	10	0.85	0.73		0.80	0.60		0.69 (0.03)	0.60 (0.00)
30	2	0.85	0.83		0.84	0.83		0.78 (0.30)	0.78 (0.20)
	6	0.86	0.81		0.85	0.83		0.79 (0.27)	0.78 (0.26)
	10	0.86	0.75		0.85	0.79		0.81 (0.35)	0.73 (0.14)
50	2	0.86	0.84		0.86	0.84		0.81 (0.83)	0.80 (0.79)
	6	0.83	0.76		0.83	0.76		0.79 (0.84)	0.72 (0.71)
	10	0.85	0.73		0.85	0.73		0.82 (0.82)	0.68 (0.59)
100	2	0.86	0.84		0.86	0.84		0.84 (0.98)	0.82 (0.97)
	6	0.85	0.78		0.85	0.79		0.82 (0.99)	0.77 (0.91)
	10	0.84	0.73		0.84	0.73		0.81 (1.00)	0.71 (0.89)
300	2	0.85	0.83		0.85	0.83		0.85 (1.00)	0.84 (1.00)
	6	0.85	0.79		0.85	0.79		0.84 (0.99)	0.80 (0.99)
	10	0.85	0.73		0.85	0.73		0.85 (1.00)	0.79 (1.00)

*Note*: “U” indicates the results for Q-matrices containing only plausible distractors. “NU” indicates the results for Q-matrices containing 2, 6, or 10 implausible distractors. “C” refers to the proportion of replications where the MC-DINA algorithm converged.

### 4.2 Study II: The effect of improper MC items on the classification of examinees

The purpose of this second simulation study was to examine to what extent the inclusion of improper items in an MC assessment affects the accuracy of the classification of examinees. However, the inclusion of improper items only affects the assignment of examinees to a few selected proficiency classes; which classes in particular depends on the specification of the q-vectors of the distractors of an improper item. Hence, the misclassification of examinees due to these improper items may be masked and hard to detect; especially, if the number of proficiency classes is large and the classification for the remaining classes is accurate. Therefore, in the second simulation study, only those proficiency classes were included that were potentially affected, given the composition of the q-vectors of the distractors of the improper items.

#### 4.2.1 Design

Similar to Study I, the different sample sizes, 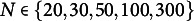 , defined layers of the experimental design, within which four levels of contamination with 1, 2, 5, and 10 improper items were nested. Like in Study I, two types of Q-matrices were created for each contamination level, 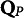 composed entirely of proper items (serving as a benchmark), and 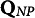 , obtained from 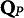 in replacing proper items by the aforementioned number of improper ones. To illustrate, the left panel of Table 6 displays five proper items that were changed into five improper ones displayed in the right panel. Notice that the five items in the right panel of Table 6 are proper for all proficiency classes except for the one having attribute profile 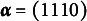 .

**Table 6:** The proper Q-matrix 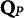 (left panel) and the corresponding improper Q-matrix 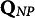 (right panel); 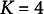 , 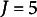

	Proper items					Improper items			
Item	A1	A2	A3	A4		A1	A2	A3	A4
1	0	0	1	1		0	0	1	1
1	1	0	1	0		1	0	1	0
1	1	1	1	0		0	1	1	0
2	0	0	1	1		0	0	1	1
2	1	1	0	0		1	1	0	0
2	1	1	1	0		0	1	1	0
3	0	0	1	1		0	0	1	1
3	1	0	0	0		1	0	0	0
3	1	1	1	0		0	1	1	0
4	0	0	1	1		0	0	1	1
4	0	0	1	0		0	0	1	0
4	1	1	1	0		1	1	0	0
5	0	0	1	1		0	0	1	1
5	1	0	1	0		1	0	1	0
5	1	1	1	0		0	1	0	0

*Note*: Notice that all items in 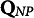 are proper for all proficiency classes except for the one having attribute profile 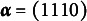 .

Imposing this specific feature across all experimental conditions (defined by the layers of sample size and the levels of item contamination nested within) required much stricter experimental control than in Study I. Thus, the 10-item version of 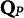 used throughout Study II was obtained in stacking the five-item Q-matrix shown in the left panel of Table 6 twice. Depending on which contamination level was required, 1, 2, 5, or 10 items in 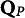 were changed resulting in the counterpart 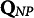 containing improper items.

Only examinees with attribute profile (1110) were included in the datasets and their responses were generated from the MC-DINA model following the same procedure used in Study I.

At each contamination level (nested within the sample size layers), a single proper Q-matrix 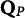 and a single improper Q-matrix 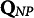 were used to generate 100 replicated datasets resulting in a total of 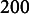 datasets per contamination level. Examinees were classified with MC-DINA and MC-NPC; the mean PAR value was computed to quantify the loss in classification.

#### 4.2.2 Results

The results of Study II are reported in Table 7. The columns under the header “ 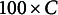 Replications” require an explanation. In a substantial number of the 100 replicated datasets, the MC-DINA algorithm encountered convergence issues such that the estimation process was disrupted and no classification result could be obtained. The proportions of these datasets are reported in parentheses (cf. columns “(C)”). In an attempt to accommodate these difficulties of MC-DINA, the PAR values for MC-NPC were re-calculated only for these replications where MC-DINA converged (cf. column “MC-DINA” under the headline “ 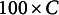 ” replications).

**Table 7:** Impact of improper items on the mean PAR values from MC-NPC and MC-DINA

		100 Replications			100 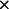 C Replications				
		MC-NPC			MC-NPC			MC-DINA	
*N*	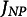	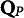	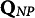		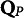	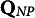		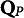 (C)	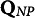 (C)
20	1	0.95	0.92		0.90	NA		0.77 (0.03)	NA (0)
	2	0.96	0.88		0.95	0.80		0.55 (0.02)	0.65 (0.03)
	5	0.96	0.64		0.91	0.59		0.67 (0.05)	0.34 (0.06)
	10	0.96	0.12		0.95	0.15		0.80 (0.03)	0.11 (0.22)
30	1	0.95	0.92		0.94	0.92		0.76 (0.21)	0.43 (0.15)
	2	0.96	0.89		0.95	0.90		0.86 (0.21)	0.43 (0.23)
	5	0.95	0.63		0.95	0.61		0.79 (0.17)	0.37 (0.29)
	10	0.95	0.12		0.92	0.13		0.77 (0.22)	0.17 (0.50)
50	1	0.96	0.92		0.96	0.92		0.80 (0.67)	0.56 (0.39)
	2	0.95	0.89		0.95	0.89		0.80 (0.69)	0.47 (0.53)
	5	0.95	0.63		0.95	0.62		0.82 (0.63)	0.39 (0.78)
	10	0.96	0.13		0.96	0.13		0.81 (0.60)	0.17 (0.81)
100	1	0.95	0.93		0.95	0.93		0.79 (0.85)	0.48 (0.76)
	2	0.95	0.89		0.95	0.89		0.80 (0.86)	0.53 (0.87)
	5	0.96	0.64		0.96	0.64		0.80 (0.84)	0.41 (0.98)
	10	0.96	0.13		0.96	0.13		0.79 (0.90)	0.18 (0.99)
300	1	0.95	0.93		0.95	0.93		0.77 (0.97)	0.56 (0.97)
	2	0.96	0.90		0.96	0.89		0.77 (0.96)	0.59 (0.98)
	5	0.95	0.62		0.95	0.62		0.78 (0.95)	0.41 (1.00)
	10	0.96	0.13		0.96	0.13		0.78 (0.94)	0.16 (1.00)

*Note*: “P” indicates the results when all distractors are proper. “NP” indicates the results when some distractors are improper. “C” identifies the proportion of replications with successful convergence.

Regardless of the sample size, the loss in examinee classification accuracy in response to an increase in the number of improper items is dramatic for MC-NPC as well as for MC-DINA. Similar to the observation made in Study I, the PAR values in case of Q-matrices not contaminated with improper items (cf. columns labeled “ 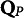 ”) remained rather stable within a specific sample size bracket for MC-NPC as well as MC-DINA. However, the overall classification accuracy for MC-DINA is significantly lower than that of MC-NPC regardless of the particular sample size.

## 5 Practical applications

### 5.1 Application I: The R function distractor.check()

In this article, the theoretical foundation of the concepts of proper and plausible MC items has been derived for data conforming to the MC-DINA model. Similarly important is the development of tailored software capable of detecting limitedly plausible and improper items automatically instead of having to inspect all MC items of a test manually one by one—a tedious and error-prone procedure. In fact, as part of this study on plausible and proper MC items, the R function distractor.check() was developed for exactly that purpose. The only input required for the distractor.check() function is the Q-matrix of the test in question. Three examples are presented to demonstrate the usage of the distractor.check() function.

The first one is taken from the article by Ozaki (2015), who developed “structured MC-DINA models” (MC-S-DINA) for remedying diagnostic ambiguity arising from improper items. Ozaki (2015) purposefully created a Q-matrix where Items 21–30 were improper, as shown in Appendix B. The distractor.check() function was used to verify that this Q-matrix, indeed, was contaminated by 10 improper items; distractor.check() produced the following output:

**Figure:**
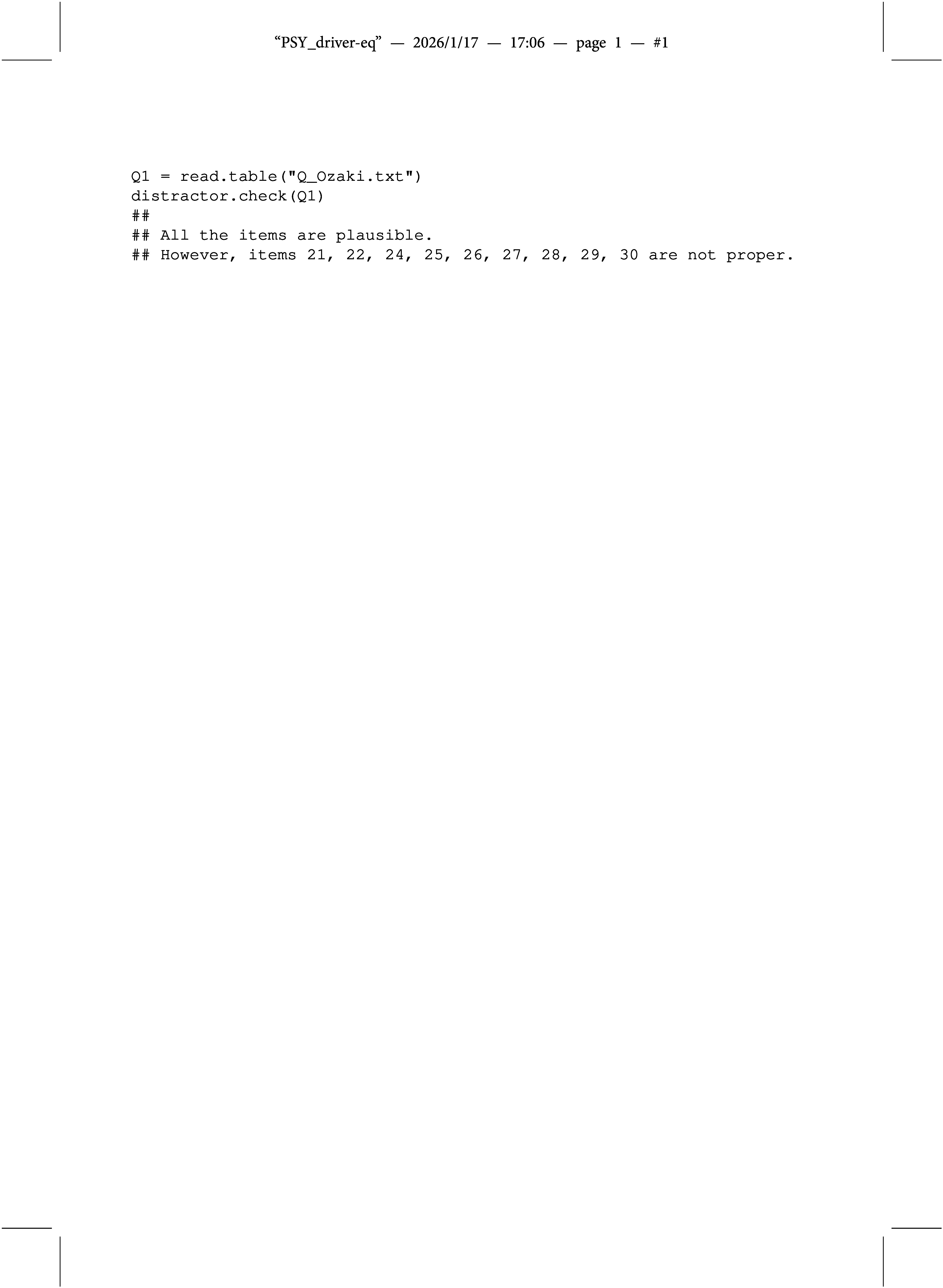

Comment: Notice that Item 23 is not listed as improper; this is not attributable to a failure of the distractor.check() function but to the fact that the two distractors of Item 23 have identical q-vectors—an anomaly that is generally inadmissible (cf. de la Torre, 2009; Wang et al., 2023). The general assumption throughout this article is that the distractors of any MC item are distinct. Hence, the distractor.check() function does not include specific checks for q-vector identity.

The second example is a Q-matrix taken from the GDINA R package. Notice that the layout of the Q-matrix required to indicate explicitly which response options are the keys.

**Figure:**
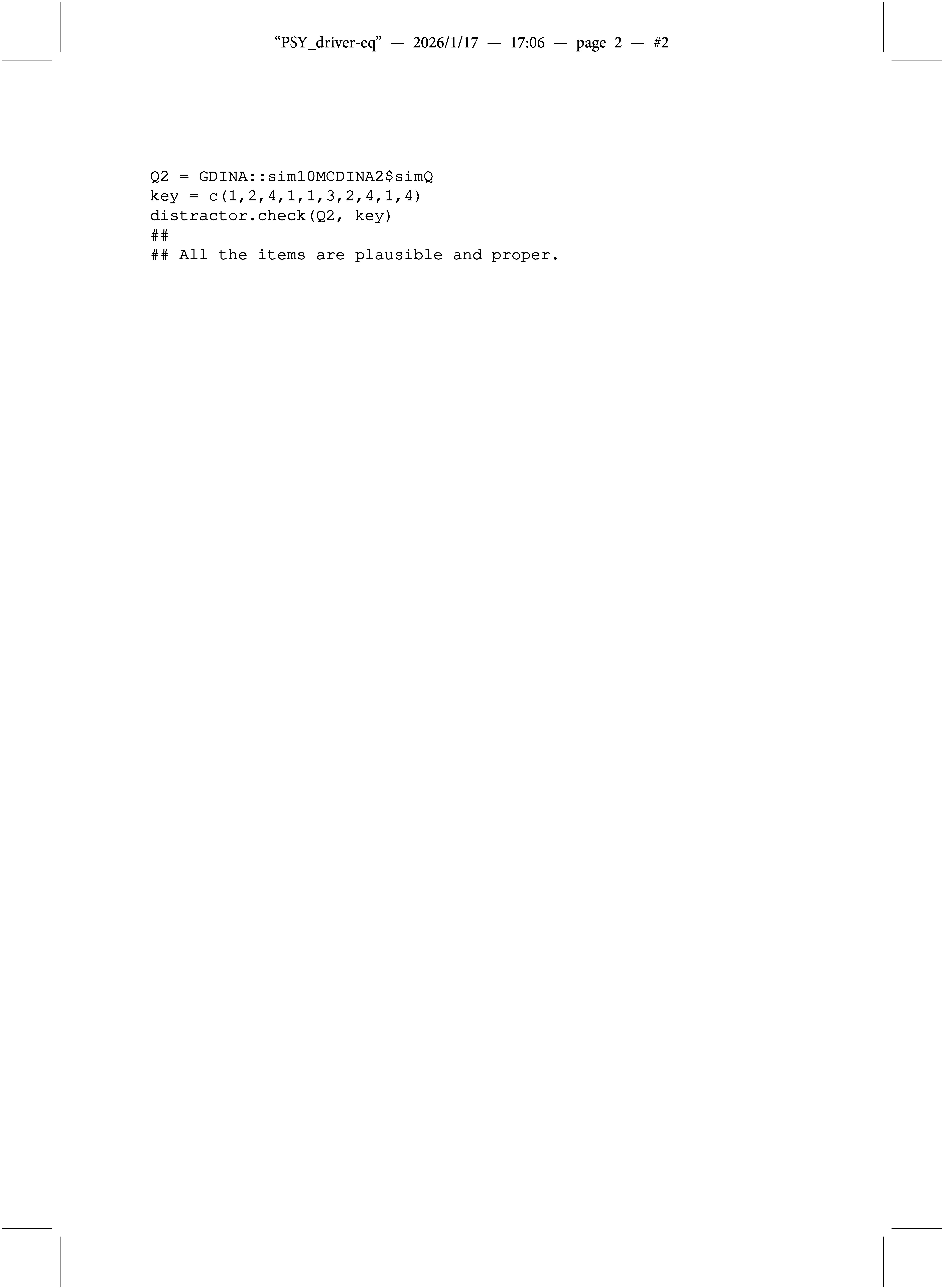

The third example is taken from a study by Wang et al. (2023), which appears to be the only published Q-matrix for MC items having coded distractors with non-nested attributes developed for “real”—non-simulated—assessment data.

**Figure:**
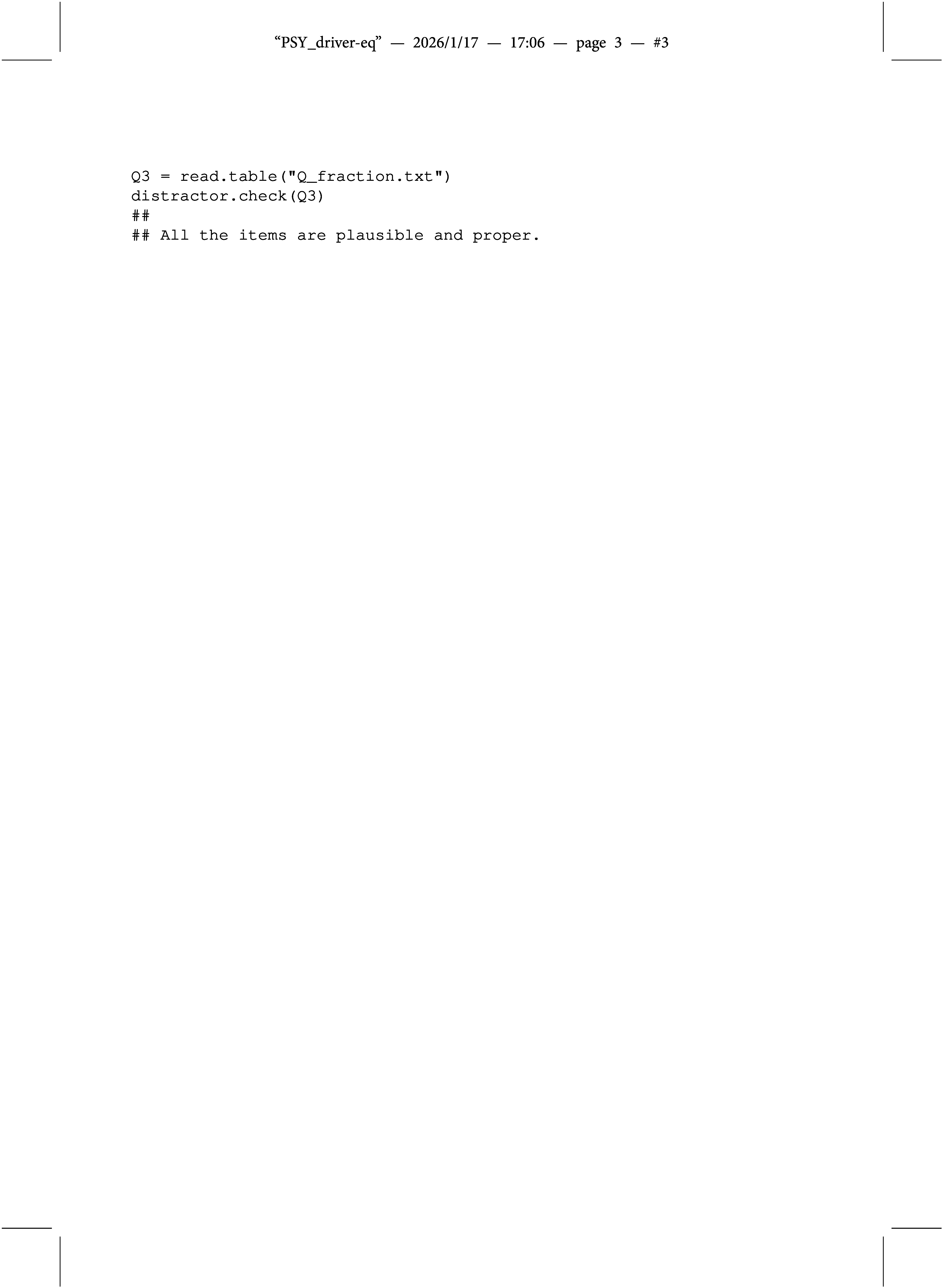

The remaining Q-matrices found in the literature were all plausible and proper because all distractors were nested within the key: de la Torre (2009); Yamaguchi (2020); Ozaki (2015), Study I; and the simulated Q-matrices in the G-DINA package. The sole exception was the Q-matrix in Ozaki’s (2015) Study II, where, as was mentioned earlier, all items were plausible but items 21–30 were improper.

### 5.2 Application II: The effect of improper items on examinee classification

For this practical application to real-world data, the dataset analyzed in Wang et al. (2023) was revisited. The data consist of responses collected from 115 examinees (after excluding cases with missing data). The test comprised 30 items designed to measure five attributes that are reported in Table 8.

**Table 8:** Attributes measured in the dataset used in Application II

Attribute	Description
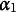	Write, read, do, and compare fractions.
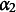	Identify and convert between proper, improper, and mixed fractions.
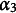	Add or subtract fractions with the same denominator.
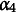	Multiply fractions.
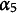	Translate a word problem into a mathematical equation.

The objective was to demonstrate the effect of improper items on examinee classification. To this end, a quasi-experimental, post hoc side-by-side analysis was conducted. First, the 30 items were screened to identify those that could be converted into improper items. Their q-vectors in the original Q-matrix were then modified accordingly. However, only Item 22 was found suitable for such modification. In its original, proper form, Item 22 had the following layout:

“Mei divides a plastic rope into 3 equal parts. Each part is 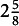 meters long. What was the original total length of the plastic rope in meters?”

The four response options with their originally coded q-vectors are: 

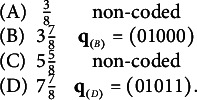

Option (D) was the key, while Option (B) was the only coded distractor. Re-examining Item 22 suggested that Option (C) could reasonably be turned into a coded distractor having q-vector 
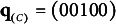
 . The rationale supporting this idea was that students who chose Option (C)—calculating 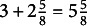 —had mastered only attribute 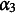 , but not the other skills required to solve Item 22 correctly. This interpretation was further supported by Item 10, which contained a distractor with the same response structure as Option (C) but was coded. Item 10 is shown below:

“ 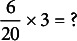 ”

The response options were 

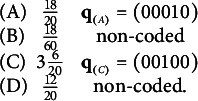

Option (A) was the key. Notice that Option (C) has the same structure as Option (C) of Item 22: 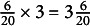 . However, whereas Option (C) of Item 22 was non-coded, Option (C) of Item 10 was coded as 
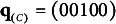
 . In both cases, though, one can argue that examinees who selected Option (C) demonstrated mastery of attribute 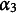 —the ability to add fractions—while failing to multiply them, a rationale consistent with that applied to Option (C) of Item 22.

Consequently, the response options for Item 22 were modified so that the q-vectors for the key and the two distractors, Options (B) and (C), became 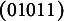 , 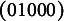 , and 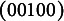 , respectively, while Option (A) remained uncoded: 

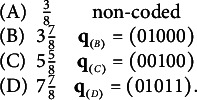

According to Theorem 2, turning Option (C) into a coded distractor makes Item 22 improper because the union of the q-vectors of Options (B) and (C) is not included among the response options. Such a modification can be justified by a general feature of the MC-DINA model: proper and plausible items with 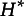 coded options classify examinees into 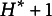 classes. Increasing the number of coded distractors broadens the spectrum of proficiency classes, leading to finer-grained classification. However, if an item is improper, it is likely to introduce ambiguity thereby decrease classification accuracy. Thus, the goal of this application was to test whether coding Option (C) of Item 22 as 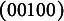 —which renders Item 22 improper—indeed results in a loss of classification accuracy.

However, with real data, assessing classification accuracy is complicated by the fact that true examinee attribute profiles are unknown. Consequently, the straightforward PAR scores used in the simulations are not applicable. To address this dilemma, for each examinee, a *K*-dimensional attribute subscore vector (Oh & Chiu, 2025; Wang et al., 2023) was calculated from their observed responses. Attribute subscores represent the proportion of items requiring a given attribute that the examinee answered correctly. These subscores were then used to assess the consistency of the estimated examinee attribute profiles: high subscores should correspond to 1 entries in 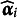 and low subscores to 0. Any discrepancy—a 1 where there should be a 0, or vice versa—indicates inconsistency between observed performance and estimated proficiency.

To isolate the impact of modifying Item 22, a shorter test of 12 items was constructed from the original 30. The subset included Items 1, 4, 9, 10, 11, 16, 18, 19, 20, 22, 23, and 28, selected according to the following criteria: (a) the Q-matrix remains complete, (b) when multiple items shared identical q-vectors, only one was retained, (c) 33% of the items included coded distractors (a proportion similar to the 30% in the original test), and (d) Item 22 was included. This dataset was analyzed using the MC-DINA model implemented in the MCmodel() function in the GDINA package, with both, the proper and the modified-improper Q-matrices. The focus was on examinees whose classification was expected to be directly affected by the improper Item 22—specifically, those who mastered attributes 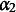 and 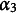 and were assigned to proficiency classes 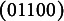 , 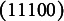 , 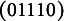 , 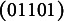 , 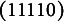 , or 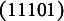 . Examinees with attribute vectors 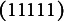 and 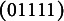 were disregarded, as they were expected to choose the key according to the MC-DINA theory.

Table 9 displays the estimated attribute profiles of the focused examinees under the two Q-matrices, alongside the subscores. Highlighted rows indicate examinees whose classification changed due to the introduction of the improper distractor.

**Table 9:** Estimated attribute profiles under the proper and improper Q-matrices and subscores; highlighted rows indicate classification changes

Proper Q-matrix						Improper Q-matrix						Subscores				
*A1*	*A2*	*A3*	*A4*	*A5*		*A1*	*A2*	*A3*	*A4*	*A5*		*A1*	*A2*	*A3*	*A4*	*A5*
0	1	1	0	1		0	1	1	0	1		0.33	0.50	0.33	0.33	0.60
1	1	1	0	1		1	1	1	0	1		1.00	0.75	0.67	0.00	0.60
1	1	1	1	0		1	1	1	1	0		1.00	0.75	0.67	0.67	0.60
1	1	1	1	0		1	1	1	1	0		1.00	0.75	0.67	0.67	0.60
0	1	1	1	0		0	1	0	1	0		0.67	0.50	1.00	0.67	0.60
0	1	1	1	0		0	1	0	1	0		0.33	0.50	1.00	0.67	0.60
1	1	1	1	0		1	1	0	1	0		1.00	0.75	1.00	0.67	0.40
1	1	1	1	0		1	1	1	1	0		0.67	0.50	0.67	0.67	0.60
0	1	1	1	0		0	1	0	1	0		0.33	0.25	0.67	0.67	0.20
1	1	1	0	0		1	1	1	0	0		1.00	0.75	0.67	0.33	0.60

As shown in Table 9, four examinees’ classifications shifted from 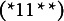 to 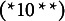 . However, three of these examinees answered all items requiring attribute 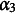 correctly based on their subscores, but they were classified as non-masters of 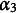 under the improper Q-matrix. This demonstrates how improper item can reduce the validity of classification results.

## 6 Discussion and conclusion

The concepts of plausible and proper MC items explore uncharted territory in CD research. Diagnostic redundancy and ambiguity were not an issue in CD assessments with MC items as long as the q-vectors of the coded distractors were constrained to be nested within the key and within each other, forming an “upside-down staircase” with the key at its top. The ordinal response scale implicitly defined by this nestedness constraint—and implemented in de la Torre’s (2009) MC-DINA model, the first CDM for MC items—ensured tight control over the diagnostic plausibility of the coded distractors of MC items. At the same time, however, the nestedness constraint imposes a serious burden on the construction of coded response options and poses challenges to assembling CD tests with MC items; especially, when large item pools are required, as in computer-adaptive testing, where item banks typically consist of hundreds or thousands of items. Relaxing the nestedness constraint therefore has clear practical benefits, but these do not come without cost: diagnostic redundancy and ambiguity in examinee classification emerge as new challenges to item and test construction.

Ozaki (2015) was apparently the first to discuss diagnostic ambiguity as a possible consequence of using MC items without the nestedness constraint. He proposed addressing this issue by modifying the parameterization of de la Torre’s MC-DINA model, thereby creating the “structured MC-DINA” (MC-S-DINA) models, which seek to remedy diagnostic ambiguity by explicit modeling it through the addition of extra parameters to de la Torre’s MC-DINA model.

The research presented in this article has a broader scope, addressing not only diagnostic ambiguity but also the presumably more prevalent issue of diagnostic redundancy. The concepts of proper and plausible MC items introduced in this article provide a theoretical framework for the technical description and analysis of both diagnostic redundancy and ambiguity. The theorems and proofs establish a solid technical foundation for the concepts of proper and plausible items, as well as strategies for remediating diagnostic redundancy and ambiguity in practice. The proposition advanced here is to redefine the set of coded distractors by modifying their q-vectors. Section Application II provides evidence from real-world data illustrating how undetected improper items can jeopardize examinee classification. In conclusion, three closely related questions remain to be addressed.

First, Simulation Study II might give the impression that diagnostic ambiguity arising from improper MC items is only a marginal problem that (i) only affects examinees in just a few proficiency classes and (ii) is typically offset by the remaining MC items in a test that are plausible and proper. The validity of this assessment certainly awaits further studies and more data. At this early stage, however, one claim can be made with certainty: diagnostic ambiguity due to improper items poses a bigger problem whenever the number of items is small, leaving too few items to compensate for improperness. Such a situation might occur in computerized adaptive testing for CD, where the selection of the next item depends on just one or two prior responses. If these happen to be improper, then the examinee is at risk of misclassification and being sent down an incorrect path. More generally, the impact of implausible and improper MC items on diagnostic accuracy in computerized adaptive testing for CD appears to be a promising avenue for future research.

Second, are diagnostic ambiguity and redundancy arising from improper and implausible MC items related to “misconceptions”—that is, the misinterpretation of the task posed by an item? DiBello et al. (2015) and Stefanutti et al. (2020, 2023) discuss in detail the structure and function of examinee misconceptions from a CD and KST point of view, respectively. They develop sophisticated strategies for modeling this intriguing phenomenon to tap into its diagnostic potential. However, diagnostic ambiguity and redundancy originate from a different source than misconceptions. The former clearly are the product of poorly constructed item–attribute vectors that do not permit for the desirable diagnostic accuracy or fail to add any additional diagnostic insights. Misconceptions, on the other hand, are persistent mistakes made by an examinee due to the erroneous interpretation of the task posed by the item. In CD, misconceptions can be detected by constructing specific q-vectors that intentionally include, for example, attribute(s) irrelevant to the task at hand, but that an examinee believes are required to solve that item. In short, diagnostic ambiguity and redundancy stem from faulty test construction, whereas misconceptions arise on the examinee’s side and can be “lured out” by crafting clever but incorrect response options. Still, since most approaches to modeling misconceptions employ an MC item setting, the question is as to what extent the proposed theorems on proper and plausible MC items might also be applicable to the study of misconceptions.

Third, many researchers in CD and KST have investigated conditions for model identifiability (e.g., Chen et al., 2015; Gu & Xu, 2019, 2021; Spoto & Stefanutti, 2020, 2023; Xu & Shang, 2018; Xu & Zhang, 2016). But what can be said about the MC-DINA model and its identifiability? Do diagnostic ambiguity and redundancy in MC items undermine model identifiability? Can theoretical concepts like proper and plausible help establish identifiability of models for MC items in CD?—The short answer to these complex questions is “not yet.” Different from identifiability of the DINA model—a well-researched topic (cf. Gu & Xu, 2021; Spoto & Stefanutti, 2020, 2023)—too little is known about the prerequisites for identifiability of the MC-DINA model, with or without the nestedness condition in place. For binary CDMs, the key requirement for identifiability is completeness of the Q-matrix (cf. Chiu et al., 2009; Gu & Xu, 2021; Köhn & Chiu, 2017). At present, conditions of Q-completeness for CDMs for MC items are unknown, in not to mention other side-conditions of identifiability as they have been established for binary CDMs. A reasonable conjecture, however, is that, in addition to the completeness of the Q-matrix, the conditions described by the concepts of proper and plausible MC items may also be necessary for establishing the identifiability of the MC-DINA model, since completeness of the Q-matrix seemingly requires the assumption of plausible and proper items. Models for MC items in CD thus open a rich field for future research. Complex topics like model identifiability should be a high priority, especially the question of whether concepts like proper and plausible can be integrated into the broader framework of model identifiability and Q-completeness, which is directly related to the assignment of examinees to their true proficiency profiles.

## Appendix A

### Proof of Theorem 1

Theorem 1. Suppose item *j* conforms to the MC-DINA model. Item *j* is plausible if and only if  for all  .
Proof Proof. (  ) Suppose item *j* is plausible. Then, according to Definition 1, the mapping  is surjective. Assume that there exists an  such that  . For those  , it is true that  due to  . Hence,  for all *k*. As a result, for these  , their ideal responses  . For the remaining  , there exists at least one *k* such that  and  . Therefore,  . In conclusion, there does not exist an  such that  , implying that the function  is not surjective, which contradicts the assumption that item *j* is plausible. Hence, if item *j* is plausible,  for all  . (  ) Suppose  for all  . To show that item *j* is plausible, the following cases are considered: Case 1:  . In this case,  . Case 2:  . In this case,  . Case 3:  , where  . Because  , or equivalently,  , there exists a *k* such that  but  . Hence, for  ,  , which implies that  . Now, because  , *l* is the highest level that  can endorse. Hence,  , which means there exists at least one  such that  . Case 4: The remaining  . Each of them will be mapped to an element in  . According to the above cases, it is true that  is surjective, implying that item *j* is plausible.

### Proof of Theorem 2

Theorem 2. Suppose item *j* conforms to the MC-DINA model. Item *j* is proper if and only if for each pair of its coded distractors with q-vectors  and  in  , it is true that  or  .
Proof. (  ) Suppose the item is proper. Assume that there exist  , where  , such that  and  . Because  , it implies that  and  as both  and  are in  . Hence,  and  . Now consider  . Suppose  . Because  , or equivalently,  , it can be concluded that examinees with attribute profile  will not choose the key and thus  , which means  and  . Note that  because  but  . Because  , there are several cases that require further discussions: Case 1:  . The item will not be proper because  , but  or equivalently,  . Case 2:  . Assume that  . Consequently,  , which contradicts the fact that  . Hence, in this case, it must be true that  . Because  but  , the item is not proper. Case 3:  . For the same rationale as that in Case 2, it is also true that  , which, again, implies that the item is not proper. Case 4:  and  . Because  , it is true that  and  . However,  and  implying that the item is not proper. Based on the above cases, the item is not proper, which contradicts to the assumption. Hence, it must be true that  or  . (  ) Suppose that for every pair of distractors  and  in  , it is true that  or  . Assume that the item is not proper. Then, there exists some  , where  , such that  and there exists a  such that  but  . Because  , it can be concluded that  . Because  , it is true that  . Because  and  , it is true that  . In addition, because  , and  ,  , where  . This is a contradiction because  . Hence,  or if there exists a  such that  , then  , which implies that the item is proper.

## Appendix B

The improper Q-matrix used in Study II in Ozaki (2015):

**Table:**

Item	Option	A1	A2	A3	A4	A5		Item	Option	A1	A2	A3	A4	A5
1	1	1	0	0	0	0		22	1	1	1	0	1	0
2	1	0	1	0	0	0		22	2	0	1	0	0	0
3	1	0	0	1	0	0		22	3	0	0	0	1	0
4	1	0	0	0	1	0		23	1	1	1	0	0	1
5	1	0	0	0	0	1		23	2	1	0	0	0	0
6	1	1	0	0	0	0		23	3	1	0	0	0	0
7	1	0	1	0	0	0		24	1	1	0	1	1	0
8	1	0	0	1	0	0		24	2	0	0	0	1	0
9	1	0	0	0	1	0		24	3	0	0	1	0	0
10	1	0	0	0	0	1		25	1	1	0	1	0	1
11	1	1	1	0	0	0		25	2	1	0	0	0	0
11	2	1	0	0	0	0		25	3	0	0	1	0	0
12	1	1	0	1	0	0		25	4	0	0	0	0	1
12	2	1	0	0	0	0		26	1	1	0	0	1	1
13	1	1	0	0	1	0		26	2	0	0	0	1	0
13	2	0	0	0	1	0		26	3	0	0	0	0	1
14	1	1	0	0	0	1		26	4	1	0	0	0	0
14	2	1	0	0	0	0		27	1	0	1	1	1	0
15	1	0	1	1	0	0		27	2	0	1	0	0	0
15	2	0	1	0	0	0		27	3	0	0	1	0	0
16	1	0	1	0	1	0		27	4	0	0	0	1	0
16	2	0	0	0	1	0		28	1	0	1	1	0	1
17	1	0	1	0	0	1		28	2	0	0	0	0	1
17	2	0	0	0	0	1		28	3	0	1	0	0	0
18	1	0	0	1	1	0		28	4	0	0	1	0	0
18	2	0	0	0	1	0		29	1	0	1	0	1	1
19	1	0	0	1	0	1		29	2	0	1	0	0	0
19	2	0	0	0	0	1		29	3	0	0	0	1	0
20	1	0	0	0	1	1		29	4	0	0	0	0	1
20	2	0	0	0	0	1		30	1	0	0	1	1	1
21	1	1	1	1	0	0		30	2	0	0	0	0	1
21	2	0	1	0	0	0		30	3	0	0	0	1	0
21	3	1	0	0	0	0		30	4	0	0	1	0	0
